# Contributions of VLDLR and LRP8 in the establishment of retinogeniculate projections

**DOI:** 10.1186/1749-8104-8-11

**Published:** 2013-06-13

**Authors:** Jianmin Su, Michael A Klemm, Anne M Josephson, Michael A Fox

**Affiliations:** 1 Virginia Tech Carilion Research Institute, Roanoke, VA 24016, USA; 2 Department of Biological Sciences, Virginia Tech, Blacksburg, VA 24061, USA; 3 Department of Anatomy and Neurobiology, Virginia Commonwealth University Medical Center, Richmond, VA 23298, USA

**Keywords:** Reelin, Synaptic targeting, Intergeniculate nucleus, Retinogeniculate, Lateral geniculate nucleus, Axon, Retinal terminal

## Abstract

**Background:**

Retinal ganglion cells (RGCs), the output neurons of the retina, project to over 20 distinct brain nuclei, including the lateral geniculate nucleus (LGN), a thalamic region comprised of three functionally distinct subnuclei: the ventral LGN (vLGN), the dorsal LGN (dLGN) and the intergeniculate leaflet (IGL). We previously identified reelin, an extracellular glycoprotein, as a critical factor that directs class-specific targeting of these subnuclei. Reelin is known to bind to two receptors: very-low-density lipoprotein receptor (VLDLR) and low-density lipoprotein receptor-related protein 8 (LRP8), also known as apolipoprotein E receptor 2 (ApoER2). Here we examined the roles of these canonical reelin receptors in retinogeniculate targeting.

**Results:**

To assess the roles of VLDLR and LRP8 in retinogeniculate targeting, we used intraocular injections of fluorescently conjugated cholera toxin B subunit (CTB) to label all RGC axons *in vivo*. Retinogeniculate projections in mutant mice lacking either VLDLR or LRP8 appeared similar to controls; however, deletion of both receptors resulted in dramatic defects in the pattern of retinal innervation in LGN. Surprisingly, defects in *vldlr*^*−/−*^;*lrp8*^*−/−*^ double mutant mice were remarkably different than those observed in mice lacking reelin. First, we failed to observe retinal axons exiting the medial border of the vLGN and IGL to invade distant regions of non-retino-recipient thalamus. Second, an ectopic region of binocular innervation emerged in the dorsomedial pole of *vldlr*^*−/−*^;*lrp8*^*−/−*^ mutant dLGN. Analysis of retinal projection development, retinal terminal sizes and LGN cytoarchitecture in *vldlr*^*−/−*^;*lrp8*^*−/−*^ mutants, all suggest that a subset of retinal axons destined for the IGL are misrouted to the dorsomedial pole of dLGN in the absence of VLDLR and LRP8. Such mistargeting is likely the result of abnormal migration of IGL neurons into the dorsomedial pole of dLGN in *vldlr*^*−/−*^;*lrp8*^*−/−*^ mutants.

**Conclusions:**

In contrast to our expectations, the development of both the LGN and retinogeniculate projections appeared dramatically different in mutants lacking either reelin or both canonical reelin receptors. These results suggest that there are reelin-independent functions of VLDLR and LRP8 in LGN development, and VLDLR- and LRP8-independent functions of reelin in class-specific axonal targeting.

## Background

Neural circuits associated with retinal ganglion cells (RGCs) have long been used as models for investigating the mechanisms that underlie circuit development and function. As the sole output neurons of the retina, RGCs must convey information regarding color, contrast, light intensity and object movement to specific regions within retino-recipient nuclei of the brain. For this reason, RGC axons project to over 20 distinct central nervous system (CNS) nuclei and are targeted to these sites by at least three distinct mechanisms. First, retinal axons are sorted topographically, so that the location of information in the visual field is accurately conveyed to a spatially correlated region of a retino-recipient nucleus [1]. Ephs/ephrins, morphogens and transmembrane cell adhesion molecules all contribute to topographic targeting of retinal axons in the mammalian brain [1-3]. In addition to being topographically mapped, retinal axons are sorted into discrete domains of most retino-recipient nuclei based upon their eye of origin. The formation of eye-specific domains requires both axonal targeting cues (such as Eph/ephrins and teneurins) [4-6], and subsequent activity-dependent refining signals (such as MHC1, C1q and neuronal pentraxins) [7-10]. The third targeting mechanism involves axons from different functional classes of RGCs targeting distinct retino-recipient nuclei or different lamina (or domains) with these nuclei. Although axonal projection patterns have recently been described for several classes of RGCs [11-19], few studies have shed light on the mechanisms underlying such class-specific targeting [20]. We recently identified reelin as a critical regulator of class-specific retinogeniculate targeting.

Reelin is a bulky, extracellular glycoprotein composed of an N-terminal f-spondin domain, eight repeated unique domains containing epidermal growth factor (EGF)-like motifs and a C-terminal domain rich in positively charged amino acids [21,22]. A multitude of studies have shown roles for reelin in neuronal migration, axonal polarization, dendritic arborization and spine formation, growth cone guidance, axonal targeting, and synaptic function and plasticity [21-30]. We previously identified reelin in a screen for synaptic targeting cues that were differentially expressed in subnuclei of the lateral geniculate nucleus (LGN): the ventral LGN (vLGN), the dorsal LGN (dLGN) and the intergeniculate leaflet (IGL) [31]. Reelin is significantly enriched in vLGN and IGL during retinogeniculate circuit formation, and mice lacking reelin (*reln*^*rl/rl*^) exhibit severe defects in retinal targeting of these regions [31]. Targeting defects in *reln*^*rl/rl*^ mutants result from the mistargeting of intrinsically photosensitive RGC (ipRGC) axons, whereas axons from other classes of RGCs appear unaffected by the absence of reelin [31].

Most functions of reelin have been attributed to its ability to bind two members of the low- density lipoprotein (LDL) receptor gene family: very-low-density lipoprotein receptor (VLDLR) and low-density lipoprotein receptor-related protein 8 (LRP8), also known as apolipoprotein E receptor 2 (ApoER2) [32,33]. Upon binding reelin, VLDLR and LRP8 activate the intracellular adaptor molecule disabled-1 (DAB1) [32]. Genetic deletion of both VLDLR and LRP8 or DAB1 result in mutant mice that outwardly resemble *reln*^*rl/rl*^ mutants [32,34-36]. Our previous studies on visual system development partially confirmed a similar molecular signaling pathway underlies reelin's function in retinal targeting: mice harboring a spontaneous mutation in DAB1 exhibit similar defects in retinogeniculate targeting as *reln*^*rl/rl*^ mutants [31]. In the present study we sought to expand these findings and test the roles of VLDLR and LRP8 in retinogeniculate targeting. We hypothesized that deletion of both canonical reelin receptors would produce defects in retinal targeting that closely resembled those in the absence of reelin. To our surprise, however, defects observed in mice lacking both VLDLR and LRP8 (*vldlr*^*−/−*^;*lrp8*^*−/−*^) were strikingly different than those in reeler mutants. Results presented here suggest that there are both reelin-independent functions of VLDLR and LRP8, and VLDLR- and LRP8-independent functions of reelin in visual system development.

## Results

### Deletion of both VLDLR and LRP8 disrupts retinogeniculate targeting

**Figure 1:**
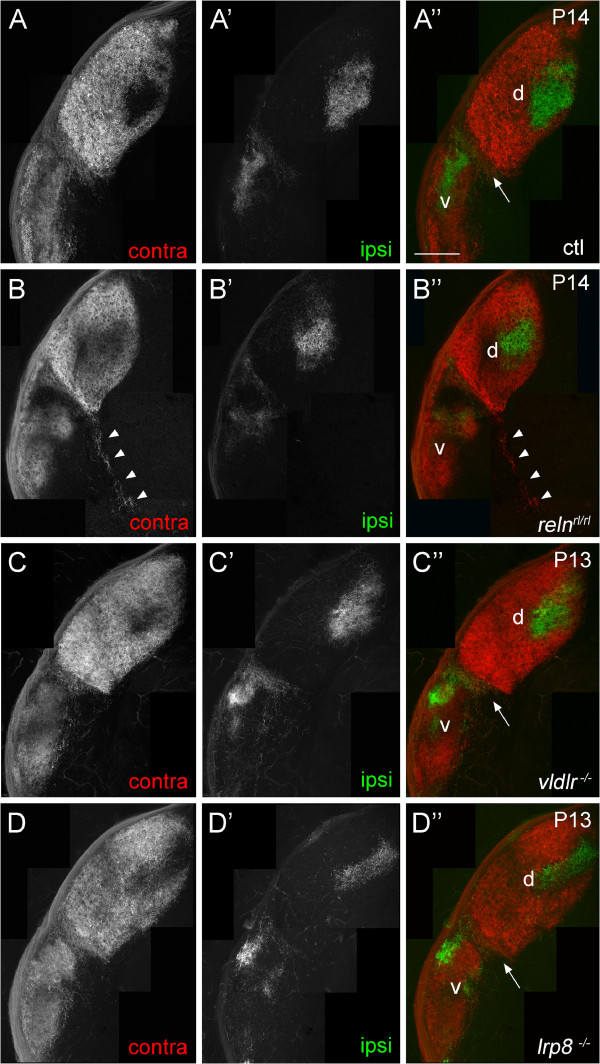
**Retinogeniculate projections in wild-type, *reln***^***rl/rl***^**, *vldlr***^***−/− ***^**and *lrp8***^***−/− ***^**mice.** Retinogeniculate projections in **(A)** P14 controls, **(B)** P14 *reln*^*rl/rl*^ mutants, **(C)** P13 *vldlr*^*−/−*^ mutants and **(D)** P13 *lrp8*^*−/−*^ mutants were labeled by intraocular injection of fluorescently conjugated CTB. Left eyes were injected with Alexa Fluor 594 CTB and right eyes were injected with Alexa Fluor 488 CTB. LGN from right hemispheres are shown. In P14 *reln*^*rl/rl*^ mutants (**B**), note the near absence of retinal projections to the IGL, and the bundle of labeled retinal axons extending out of the LGN and into non-retino-recipient thalamus (arrowheads). ‘Contra’ denotes projections originating from the contralateral retina and ‘ipsi’ denotes projections originating from the ipsilateral retina. dLGN are labeled ‘d’, vLGN are labeled ‘v’ and arrows indicate IGL. The color of the label in panels (**A** to **D**) and (**A’** to **D’**) indicates the color of the image shown in (**A”** to **D”**). Scale bar = 200 μm. CTB, cholera toxin B subunit; dLGN, dorsal LGN; IGL, intergeniculate leaflet; LGN, lateral geniculate nucleus; vLGN, ventral LGN.

To assess the roles of VLDLR and LRP8 in retinogeniculate targeting we analyzed retinal projections in *vldlr*^−/−^ and *lrp8*^−/−^ mutant mice. These mice are viable, fertile, and their appearance and cage behavior are indistinguishable from wild-type and heterozygote littermates. Intraocular injections of fluorescently conjugated cholera toxin B subunit (CTB) were used to label all RGC axons. Axonal projections were analyzed during the second and third postnatal weeks of mouse development, a period of development when retinal axons have fully innervated the dLGN, vLGN and IGL (Figure 1A). Projections from each eye were labeled with distinct fluorescently conjugated versions of CTB, so that eye-specific projections could be distinguished in the LGN. In contrast to mutants lacking reelin (Figure 1B), the gross pattern of retinal projections to each LGN subnuclei in mutants lacking either VLDLR or LRP8 appeared similar to those observed in littermate controls in many regards (Figure 1C,D). Specifically, retinal axons fully arborized in the IGL, and no retinal axons were observed exiting the medial border of the vLGN or IGL and invading non-retino-recipient regions of medial thalamus (Figure 1C,D and see arrowheads in 1B) (Table 1). Although these gross patterns of retinal innervation in *vldlr*^−/−^ and *lrp8*^−/−^ mutant mice appeared similar to littermate controls, the absence of these receptors (and reelin) clearly affected activity-dependent refinement of retinal projections into fully segregated, eye-specific domains in both vLGN and dLGN.

**Table 1:** Retinogeniculate projection in phenotypes in wild-type and mutant mice

**Genotype**	**Smaller retino-recipient region of vLGN**	**Sparse retinal projections to IGL**	**Misrouted retinal axons exiting medial border of LGN**	**Ectopic retinal projections in dLGN**
Control	-	-	-	-
	(0/11)	(0/11)	(0/11)	(0/11)
*reln*^*rl/rl*^	**++**	++	++	-
	7/7	(7/7)	(7/7)	(0/7)
*vldlr*^*−/−*^	-	-	-	+/−
	(0/11)	(0/11)	(0/11)	(2/11)
*lrp8*^*−/−*^	-	-	-	-
	(0/9)	(0/9)	(0/9)	(0/9)
*vldlr*^*+/−*^;*lrp8*^*+/−*^	-	-	-	-
	(0/9)	(0/9)	(0/9)	(0/9)
*vldlr*^*+/−*^;*lrp8*^*−/−*^	-	+	-	+
	(0/6)	(2/6)	(0/6)	(2/6)
*vldlr*^*−/−*^;*lrp8*^*+/−*^	+/−	+	+/−	+
	(4/7)	(3/7)	(1/7)	(5/7)
*vldlr*^*−/−*^;*lrp8*^*−/−*^	++	++	+/−	++
	(7/7)	(7/7)	(2/7)	(7/7)

The presence of four distinct defects in retinogeniculate projection patterns was assessed in various mutant mice: 1) small retinal projection patterns to vLGN; 2) a region of sparse retinal projections in or adjacent to IGL; 3) retinal axons misrouted out of the medial border of the vLGN and IGL; and 4) an ectopic region of binocular retinal projections in the dorsomedial pole of dLGN. Phenotypes were scored as being: −, absent; +/−, weak and only occasionally present; +, strong but only occasionally present; and ++, strong and highly penetrant. Parentheses indicate the number of mice exhibiting a phenotype compared to the total number of mice analyzed for that genotype. All mice included in these analyses ranged in age from P9 to P23. dLGN, dorsal LGN; IGL, intergeniculate leaflet; LGN, lateral geniculate nucleus; vLGN, ventral LGN.

**Figure 2:**
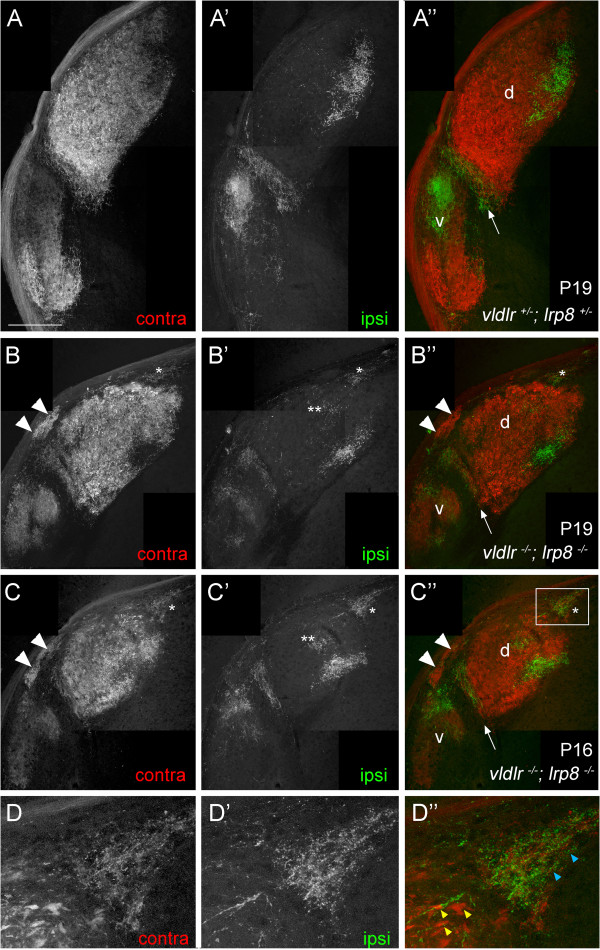
**Deletion of both VLDLR and LRP8 results in dramatic defects in retinogeniculate projections.** Retinogeniculate projections in **(A)** P19 control, **(B)** P19 *vldlr*^*−/−*^;*lrp8*^*−/−*^ double mutant and **(C)** P16 *vldlr*^*−/−*^;*lrp8*^*−/−*^ double mutant mice were labeled by intraocular injection of fluorescently conjugated CTB. In *vldlr*^*−/−*^;*lrp8*^*−/−*^ double mutants (**B**,**C**), note the absence of projection between the IGL and vLGN, the ectopic clusters of retinal arbors adjacent to the optic tract (arrowheads), and the ectopic region of binocular retinal input in the dorsomedial pole of the dLGN (asterisks). Double asterisks (**) indicate regions where retinal arbors from the ipsilateral retina have not been fully refined into segregated, eye-specific domains. **(D)** High magnification views of the boxed region in (**C**). Blue and yellow arrowheads in (**D**) highlight differences in retinal terminal size in dLGN (yellow) and the ectopic region at the dorsomedial pole of dLGN (blue). Left eyes were injected with Alexa Fluor 594 CTB and right eyes were injected with Alexa Fluor 488 CTB. LGN from right hemispheres are shown. ‘Contra’ denotes projections originating from the contralateral retina and ‘ipsi’ denotes projections originating from the ipsilateral retina. dLGN are labeled ‘d’, vLGN are labeled ‘v’ and arrows indicate IGL. The color of the label in panels (**A** to **D**) and (**A’** to **D’**) indicates the color of the image shown in (**A”** to **D”**). Scale bar = 200 μm for (**A** to **C**) and 75 μm for (**D**). CTB, cholera toxin B subunit; dLGN, dorsal LGN; IGL, intergeniculate leaflet; LGN, lateral geniculate nucleus; LRP8, low-density lipoprotein receptor-related protein 8; VLDLR, very-low-density lipoprotein receptor; vLGN, ventral LGN.

Previous studies have demonstrated that VLDLR and LRP8 are capable of compensating for each other [32]. For this reason, we explored whether deletion of both VLDLR and LRP8 affects retinogeniculate targeting. *Vldlr*^*−/−*^;*lrp8*^*−/−*^ double mutant mice are born in expected ratios and are indistinguishable from littermate controls (or *vldlr*^−/−^ or *lrp8*^−/−^ single mutants) at birth. By the end of the first week of postnatal development, *vldlr*^*−/−*^;*lrp8*^*−/−*^ mutants appeared smaller than littermates and by postnatal day 12 (P12) double mutants exhibited an ataxic gait (similar to *reln*^*rl/rl*^ mutants). Retinal projections were labeled by intraocular injection of CTB in P9 to P23 *vldlr*^*−/−*^;*lrp8*^*−/−*^ mutants (n = 6). Several defects in retinal projections in the absence of both VLDLR and LRP8 appeared similar to defects observed in mutants lacking reelin (Figure 1B and 2B,C). Specifically, the spatial extent of retinal innervation to vLGN and IGL appeared markedly decreased in *vldlr*^*−/−*^;*lrp8*^*−/−*^ mutants (Figure 2B,C). Additionally, a region that lacked retinal arbors appeared to separate the vLGN and IGL in *vldlr*^*−/−*^;*lrp8*^*−/−*^ mutants (Figure 2B,C). Despite these similarities with retinogeniculate targeting in the absence of reelin, we were surprised to find that a well-defined set of retinal projections to the IGL persisted in *vldlr*^*−/−*^;*lrp8*^*−/−*^ mutants and very few retinal axons were observed exiting the medial border of the double mutant vLGN or IGL (Figure 2B,C and Table 1). In the few *vldlr*^*−/−*^;*lrp8*^*−/−*^ mutants (two out of six) where such misrouted axons were observed they were seen only in a small number of coronal LGN sections and were typically single axons that extended less than 100 μm from the LGN. These rare misrouted axons differed dramatically from the consist bundles of misrouted axons observed in *reln*^*rl/rl*^ mutants that extend for several hundred microns outside of the LGN (see Figure 1B).

**Figure 3:**
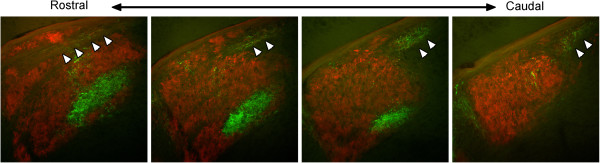
**Retinogeniculate projections in rostral and caudal sections of *vldlr***^***−/−***^**;*lrp8***^***−/− ***^**dLGN.** Retinogeniculate projections in a P16 *vldlr*^*−/−*^;*lrp8*^*−/−*^ mutant were labeled by intraocular injection of fluorescently conjugated CTB. The left eye was injected with Alexa Fluor 594 CTB and the right eye was injected with Alexa Fluor 488 CTB. Several sections of the same LGN from rostral to caudal are shown. Projections originating from the contralateral retina are labeled in red and projections originating from the ipsilateral retina are labeled in green. Arrowheads indicate regions of binocular, abnormal retinal projections. In central and caudal LGN sections, this region of ectopic innervation is in the dorsomedial pole of mutant dLGN. However, in extreme caudal sections, the ectopic innervation invades more lateral dLGN regions. Scale bar = 200 μm. CTB, cholera toxin B subunit; dLGN, dorsal LGN; LGN, lateral geniculate nucleus.

Unexpectedly, two defects were observed in retinal projections in *vldlr*^*−/−*^;*lrp8*^*−/−*^ mutants that we failed to observe in the >20 *reln*^*rl/rl*^ mutants we have previously analyzed. First, dense patches of retinal arbors originating from the contralateral eye were directly adjacent to, and in some cases embedded within, the optic tract (see arrowheads in Figure 2B,C). Second, an ectopic patch of retinal arbors was present in the dorsomedial pole of dLGN (see asterisks in Figure 2B,C). Although this region of ectopic innervation was most commonly observed in the dorsomedial pole of mutant dLGN, it appeared to invade a more lateral portion of dLGN in extreme rostral sections of mutant LGN (Figure 3). Retinal arbors in these ectopic regions of innervation originated from both contra- and ipsilateral retinas, and were not segregate into eye-specific domains.

In the process of generating *vldlr*^*−/−*^;*lrp8*^*−/−*^ mutants, a large number of single reelin receptor gene mutants were generated that carried only one copy of the other canonical reelin receptor (*vldlr*^−/−^;*lrp8*^+/−^ and *vldlr*^+/−^;*lrp8*^−/−^ mice). We analyzed retinal projections in these mice to test the affect of gene dosage of these receptors on retinal targeting. All of the defective phenotypes described above for double mutants were observed in mutant heterozygote mice, although phenotypes were typically not as dramatic and not as penetrant as in double mutants (Table 1).

### Deletion of both VLDLR and LRP8 misroutes retinal axons to the dorsomedial pole of dLGN

**Figure 4:**
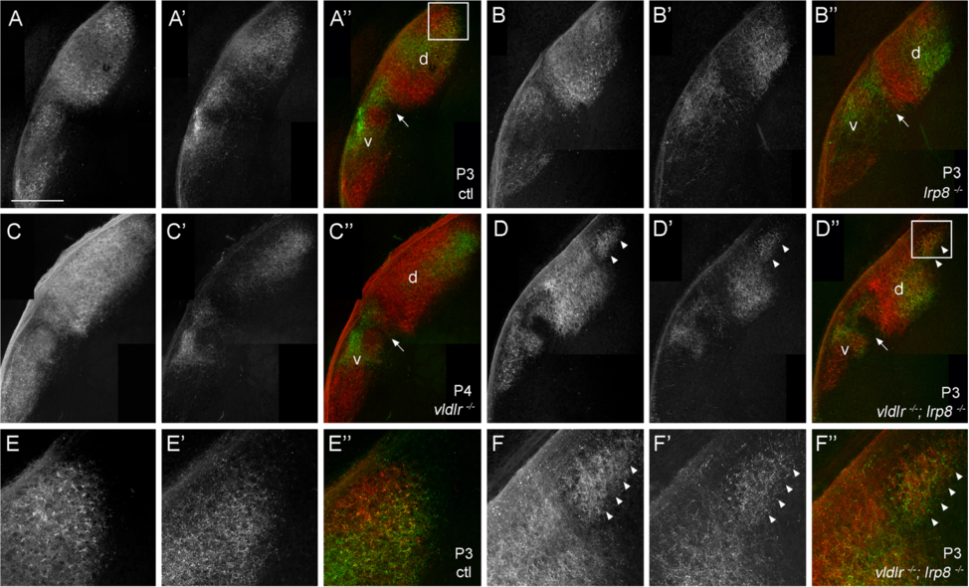
**Development of retinogeniculate projections in wild-type, *vldlr***^***−/−***^**,*lrp8***^***−/− ***^**and *vldlr***^***−/−***^**;*lrp8***^***−/− ***^**mice.** Retinogeniculate projections in **(A)** P3 controls, **(B)** P3 *lrp8*^*−/−*^ mutants, **(C)** P4 *vldlr*^*−/−*^ mutants and **(D)** P3 *vldlr*^*−/−*^;*lrp8*^*−/−*^ mutants were labeled by intraocular injection of fluorescently conjugated CTB. In (**D**), note the ectopic region of binocular retinal input in the dorsomedial pole of *vldlr*^*−/−*^;*lrp8*^*−/−*^ dLGN is labeled with arrowheads. **(E,F)** High magnification images of the areas indicated by boxes in (**A**) and (**D**), respectively. Left eyes were injected with Alexa Fluor 594 CTB and right eyes were injected with Alexa Fluor 488 CTB. LGN from right hemispheres are shown. ‘Contra’ denotes projections originating from the contralateral retina and ‘ipsi’ denotes projections originating from the ipsilateral retina. dLGN are labeled ‘d’, vLGN are labeled ‘v’ and arrows indicate IGL. The color of the label in panels (**A** to **D**) and (**A’** to **D’**) indicates the color of the image shown in (**A”** to **D”**). Scale bar = 200 μm for (**A** to **D**) and 75 μm for (**E**,**F**). CTB, cholera toxin B subunit; dLGN, dorsal LGN; IGL, intergeniculate leaflet; LGN, lateral geniculate nucleus; vLGN, ventral LGN.

We next set out to understand the mechanisms that underlie the formation of the ectopic, binocularly innervated region of dorsomedial dLGN in *vldlr*^*−/−*^;*lrp8*^*−/−*^ mutants. At perinatal ages (P3 to P4) projections from both eyes form diffuse, overlapping arbors in wild-type dLGN and vLGN. Similar diffuse projections were observed from ipsi- and contralateral retinas in *vldlr*^−/−^ mutants, *lrp8*^−/−^ mutants, and *vldlr*^*−/−*^;*lrp8*^*−/−*^ double mutants (Figure 4). Two striking defects were observed in retinal projections at perinatal ages in *vldlr*^*−/−*^;*lrp8*^*−/−*^ mutants. First, a lack of arbors was observed in the region separating the IGL and vLGN, similar to projections in *reln*^*rl/rl*^ mutants at this age (although no axons were observed exiting the medial border of vLGN and IGL in *vldlr*^*−/−*^;*lrp8*^*−/−*^ mutants (n = 3)). Second, retinal axons appeared to innervate a distinct patch of thalamus that was dorsal to the dLGN in *vldlr*^*−/−*^;*lrp8*^*−/−*^ mutants (Figure 4D,F). Projections to this ectopic region originated from both ipsi- and contralateral retinas. These findings supported the notion that retinal axons were initially mistargeted into the dorsomedial pole of dLGN in *vldlr*^*−/−*^;*lrp8*^*−/−*^ mutants.

**Figure 5:**
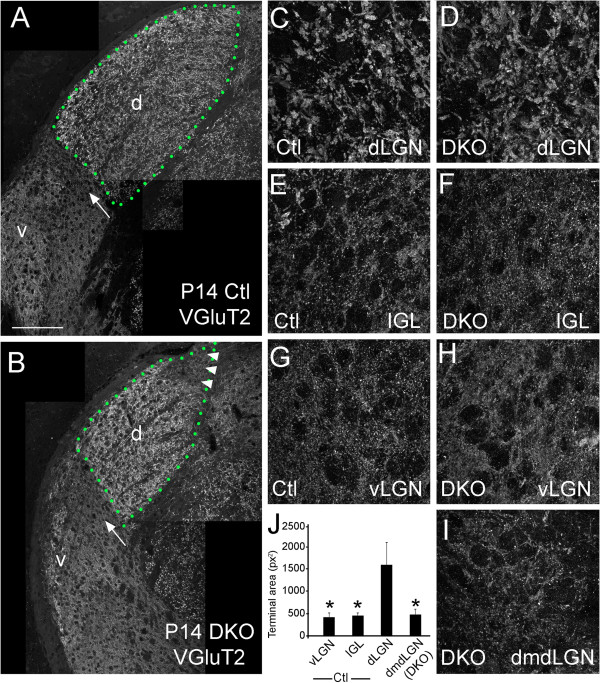
**Retinal terminals are smaller in the dorsomedial pole of *vldlr***^***−/−***^**;*lrp8***^***−/− ***^**dLGN than throughout the rest of the dLGN.** Retinal terminals were labeled in **(A)** P14 control and **(B) ***vldlr*^*−/−*^;*lrp8*^*−/−*^ mutant (DKO) LGN by VGluT2-immunostaining. In (**B**), note the ectopic region of binocular retinal input in the dorsomedial pole of *vldlr*^*−/−*^;*lrp8*^*−/−*^ dLGN is labeled with arrowheads. **(C to I)** High magnification images of VGluT2-labeled terminals in control and mutant dLGN, IGL and vLGN. VGluT2-immunolabeled terminals in the dorsomedial pole of mutant dLGN (dmdLGN) are shown in (**I**). Note that the size of VGluT2-containing terminals in dorsomedial pole of mutant dLGN are dramatically smaller than those in dLGN (**C**,**D**) and instead appear similar to terminals in vLGN and IGL (**E**,**F**). **(J)** Terminals sizes were measured in ImageJ. Terminal areas (in pixels^2^) were significantly larger in dLGN than in vLGN or IGL in controls. Terminal sizes in dorsomedial pole of mutant dLGN of DKO appeared quantitatively similar to control vLGN and IGL. *P* <0.05 for terminal sizes in vLGN (control), IGL (control) and dorsomedial pole of mutant dLGN (DKO) compared with dLGN (control) by ANOVA. Differences in terminal size between vLGN (control), IGL (control) and dorsomedial pole of mutant dLGN (DKO) were not statistically significant. The outline of the dLGN is depicted with green dots. dLGN are labeled ‘d’, vLGN are labeled ‘v’ and arrows indicate IGL. Scale bar = 160 μm for (**A**,**B**) and 30 μm for (**C** to **I**). Ctl, control; DKO, double knockout; dLGN, dorsal LGN; dmdLGN, dorsomedial pole of mutant dLGN; IGL, intergeniculate leaflet; LGN, lateral geniculate nucleus; VGluT2, vesicular glutamate transporter 2; vLGN, ventral LGN.

Further support for the idea that retinal axons were misrouted into the dorsomedial pole of dLGN in double mutants came from two observations regarding the morphology of CTB-labeled terminals in the ectopic patch of retinal innervation in dLGN. First, as noted above, retinal arbors appeared unsegregated based upon their eye of origin, a feature normally common only to those arbors within the IGL. Second, terminals in the dorsomedial pole of double mutant dLGN appeared morphologically smaller than other retinal terminals in dLGN (Figure 2D). We verified this result by labeling retinal terminals with antibodies against vesicular glutamate transporter 2 (VGluT2), a synaptic vesicle-associated protein whose distribution in LGN is restricted to retinal terminals [37]. Importantly, VGluT2 is specifically enriched in terminals and is largely absent from non-synaptic regions of axons. VGluT2-immunostaining revealed that retinal terminals in wild-type dLGN were considerably larger than their counterparts in vLGN and IGL (Figure 5A); and, as noted previously [38], the intensity of VGluT2-immnoreactivity was considerably less robust in wild-type IGL than in the adjacent vLGN and dLGN (Figure 5A). Similar differences in terminal size were observed in retinal terminals in vLGN, IGL and dLGN in *vldlr*^*−/−*^;*lrp8*^*−/−*^ mutants (Figure 5B). Analysis of the ectopic region of retinal innervation in the dorsomedial pole of *vldlr*^*−/−*^;*lrp8*^*−/−*^ mutant dLGN revealed terminal size was morphologically and quantitatively similar to terminals in control vLGN and IGL rather than those in the dLGN (Figure 5C to J). Taken together with developmental analyses, these data suggest that the ectopic region of retinal innervation in *vldlr*^*−/−*^;*lrp8*^*−/−*^ mutants resulted from defects in axonal targeting and not from aberrant retinal refinement.

### Cytoarchitectural defects in the LGN of *vldlr*^*−/−*^;*lrp8*^*−/−*^ mutants

**Figure 6:**
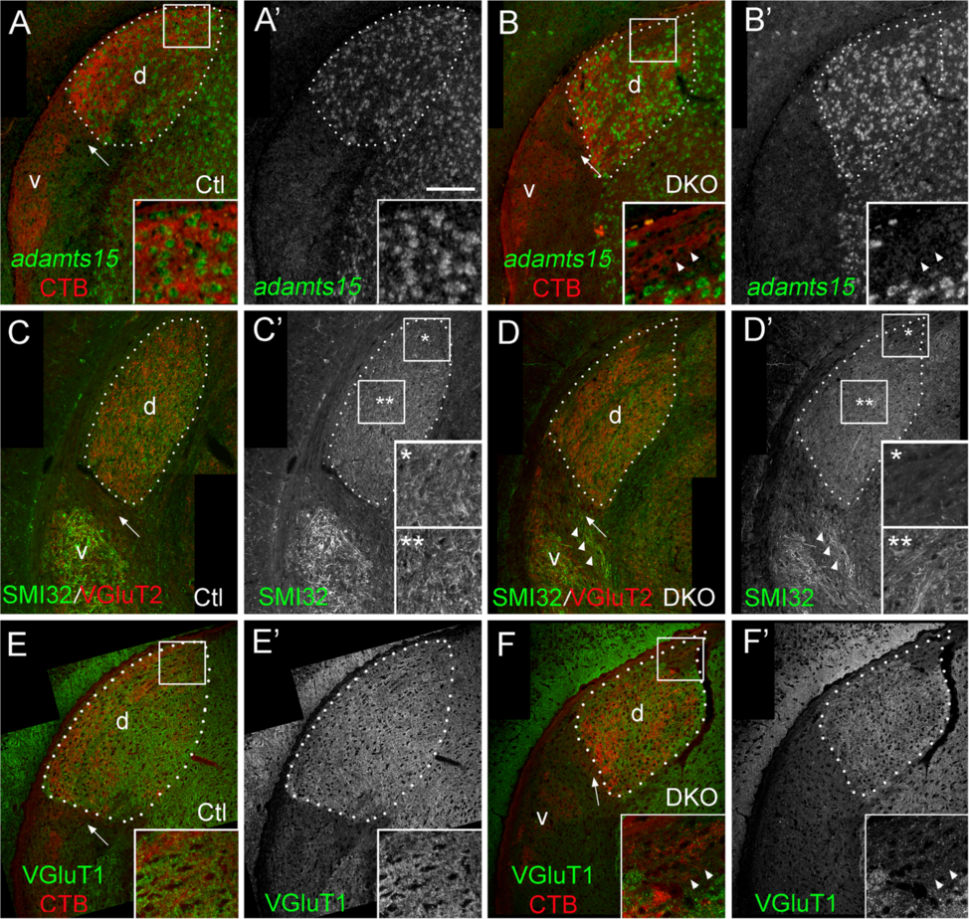
**Abnormal cytoarchitecture of the dorsomedial pole of *vldlr***^***−/−***^**;*lrp8***^***−/− ***^**mutant dLGN. ***Adamts15* mRNA, which is expressed by thalamic relay neurons, was labeled by *in situ* hybridization in **(A)** P14 control and **(B)***vldlr*^*−/−*^;*lrp8*^*−/−*^ mutant (DKO) LGN. Retinal arbors were labeled by binocular injection of Alexa Fluor 594 CTB. In (**B**), note the ectopic region of binocular retinal input in the dorsomedial pole of *vldlr*^*−/−*^;*lrp8*^*−/−*^ dLGN is labeled with arrowheads. Insets in (**A**,**B**) show high magnification images of the areas indicated by boxes. **(C,D)** dLGN and vLGN relay neurons (and their dendrites) were labeled by SMI32-immunostaining and retinal terminals were labeled by VGluT2-IHC. Note the absence of SMI32-immunoreactivity in the dorsomedial pole of *vldlr*^*−/−*^;*lrp8*^*−/−*^ mutant dLGN (asterisk). Also note the abnormal presence of SMI32-IR structures in *vldlr*^*−/−*^;*lrp8*^*−/−*^ mutant IGL (arrowheads). Insets in (**C**,**D**) show high magnification images of the areas indicated by boxes, and single and double asterisks. **(E,F)** Corticothalamic terminals in control and mutant dLGN were labeled by VGluT1-IHC. Retinal arbors were labeled by binocular injection of Alexa Fluor 594 CTB. In (**F**), note the absence of VGluT1-immunoreactivity in the dorsomedial pole of *vldlr*^*−/−*^;*lrp8*^*−/−*^ mutant dLGN (arrowheads). Insets in (**E**,**F**) show high magnification images of the areas indicated by boxes. For all images, the outline of the dLGN is depicted with white dots. dLGN are labeled ‘d’, vLGN are labeled ‘v’ and arrows indicate IGL. Scale bar = 180 μm for (**A** to **F**) and 50 μm for insets. CTB, cholera toxin B subunit; Ctl, control; DKO, double knockout; dLGN, dorsal LGN; IGL, intergeniculate leaflet; LGN, lateral geniculate nucleus; SMI32-IR, SMI32-immunoreactivity; VGluT1-IHC, vesicular glutamate transporter 1-immunohistochemistry; vLGN, ventral LGN.

We next sought to understand if cytoarchitectural changes in the LGN of *vldlr*^*−/−*^;*lrp8*^*−/−*^ mutants might underlie mistargeting of retinal axons into the dorsomedial pole of dLGN. For this we assessed the expression of *a d*isintegrin *a*nd *m*etalloproteinase with *t*hrombo*s*pondin motif 15 (ADAMTS15). We previously identified *adamts15* mRNA as a reliable marker of dLGN neurons [31]. *In situ* hybridization confirmed the enrichment of *adamts15* mRNA in wild-type dLGN neurons, and its absence from vLGN and IGL (Figure 6A). In *vldlr*^*−/−*^;*lrp8*^*−/−*^ mutants, *adamts15* mRNA was similarly absent from vLGN and IGL, and enriched throughout much of the dLGN (Figure 6B). Importantly, however, it appeared absent from the dorsomedial pole of the mutant dLGN where ectopic retinal arbors were present (Figure 6B). This suggested that dLGN relay neurons were absent from this region of mutant dLGN. We confirmed these results with an antibody directed against non-phosphorylated neurofilaments (SMI32), which has previously been shown to specifically label relay neurons and their dendrites in mouse dLGN and vLGN (but not in IGL) [31,39]. Immunostaining of wild-type P14 LGN produced a characteristic label of dLGN and vLGN neuropil (Figure 6C). In *vldlr*^*−/−*^;*lrp8*^*−/−*^ mutants similar reactivity was noted, with two exceptions: 1) little, if any, SMI32-immunoreactivity (SMI32-IR) was observed in the dorsomedial pole of the double mutant dLGN (Figure 6D, see single asterisk); and 2) SMI32-IR neurons were observed extending into the medial border of the double mutant IGL (Figure 6D, see arrowhead).

To further confirm cytoarchitectural differences in the dorsomedial pole of *vldlr*^*−/−*^;*lrp8*^*−/−*^ mutant dLGN, we next assessed the presence of other types of nerve terminals present in dLGN. A large portion of inputs to dLGN arise from corticothalamic (CT) axons originating from layer VI of visual cortex [40]. Terminals from these CT axons contain the synaptic vesicle associated protein, vesicular glutamate transporter 1 (VGluT1) [37,41]. While dense populations of VGluT1-containing terminals were present throughout the dorsal thalamus and dLGN of *vldlr*^*−/−*^;*lrp8*^*−/−*^ mutants, they appeared absent from the dorsomedial pole of mutant dLGN (Figure 6F). Taken together, studies on the distribution of *adamts15* mRNA, SMI32-IR and VGluT1-containing terminals all suggest that the dorsomedial pole of *vldlr*^*−/−*^;*lrp8*^*−/−*^ mutant dLGN is cytoarchitecturally different than adjacent regions of dLGN.

**Figure 7:**
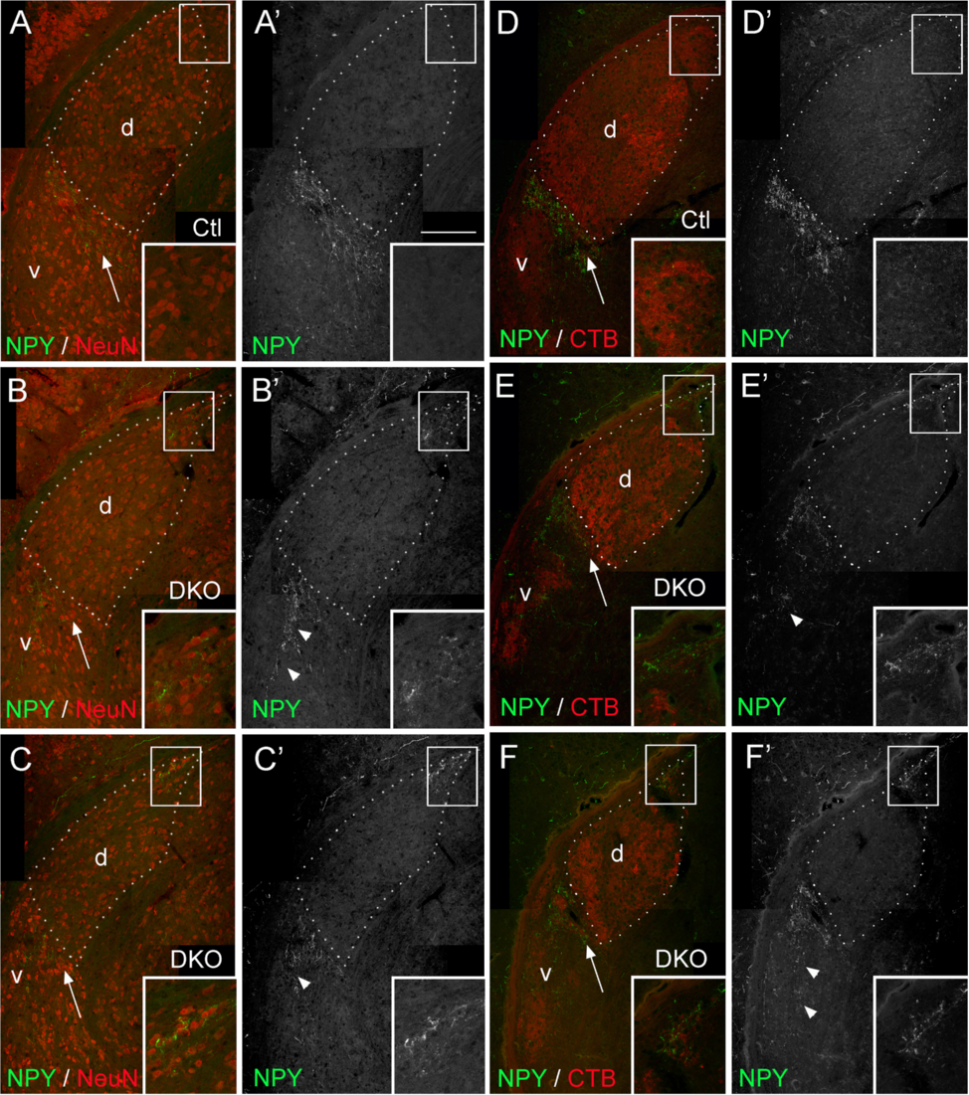
**Malposition of IGL neurons in *vldlr***^***−/−***^**;*lrp8***^***−/− ***^**mutant LGN. (A,B)** NPY-expressing neurons, which are normally restricted to the IGL, were immunolabeled in **(A,D)** P14 control and **(B,C,E,F)***vldlr*^*−/−*^;*lrp8*^*−/−*^ mutant (DKO) LGN. In (**A** to **C**), LGN neurons were colabeled by NeuN-IHC. In (**D** to **F**), retinal arbors were colabeled by binocular injection of Alexa Fluor 594 CTB. Insets show high magnification images of the boxed areas in (**A** to **F**). Note the presence of NPY-immunoreactivity in the dorsomedial pole of *vldlr*^*−/−*^;*lrp8*^*−/−*^ mutant dLGN (as seen in insets). NPY-immunoreactivity also extended into the vLGN in *vldlr*^*−/−*^;*lrp8*^*−/−*^ mutant LGN (arrowheads). For all images, the outline of the dLGN is depicted with white dots. dLGN are labeled ‘d’, vLGN are labeled ‘v’ and arrows indicate IGL. Scale bar = 180 μm for (**A** to **F**) and 50 μm for insets. CTB, cholera toxin B subunit; DKO, double knockout; dLGN, dorsal LGN; IGL, intergeniculate leaflet; IHC, immunohistochemistry; LGN, lateral geniculate nucleus; Neu-N; neuronal nuclei; NPY, neuropeptide Y; vLGN, ventral LGN.

Since dLGN relay neurons appeared absent from the dorsomedial pole of mutant dLGN, we next asked whether any neurons were present in this region. Indeed, neuronal nuclei (NeuN) immunolabeling (which labels most neurons in mouse brain) revealed a normal density of neurons in mutant dLGN, including its dorsomedial pole (Figure 7A,B,C). Since neurons in the mutant dorsomedial pole lacked relay neuron markers we asked what their identity might be. First, we applied riboprobes against synaptotagmin 2 mRNA (*syt2*), a marker of vLGN neurons [31], to assess whether neurons in the dorsomedial pole of mutant dLGN may be derived from the vLGN. No *syt2*-expressing neurons were detected in the dorsomedial pole of mutant dLGN (data not shown). Next, we addressed whether these displaced neurons might be from the IGL, a possibility that seemed reasonable since IGL neurons do not express *adamts15* mRNA and are not labeled by SMI32 antibodies [31]. Additionally, aberrant retinal projections in the mutant dorsomedial dLGN pole fail to segregate into eye-specific domains and generate relatively small terminals, both features of retinal inputs to the IGL. To test our hypothesis we assessed the distribution of neuropeptide Y (NPY) in *vldlr*^*−/−*^;*lrp8*^*−/−*^ mutant LGN. In wild-type LGN, NPY-expressing neurons are present in the IGL and are absent from all other adjacent thalamic regions (Figure 7A,D). In *vldlr*^*−/−*^;*lrp8*^*−/−*^ mutants, NPY-expression was present in IGL (although it appeared less organized than in controls), but was also present in vLGN and in the dorsomedial pole of the dLGN (Figure 7B,C,E,F). These results reveal that IGL neurons are malpositioned in the dorsomedial pole of the dLGN in *vldlr*^*−/−*^;*lrp8*^*−/−*^ mutants.

**Figure 8:**
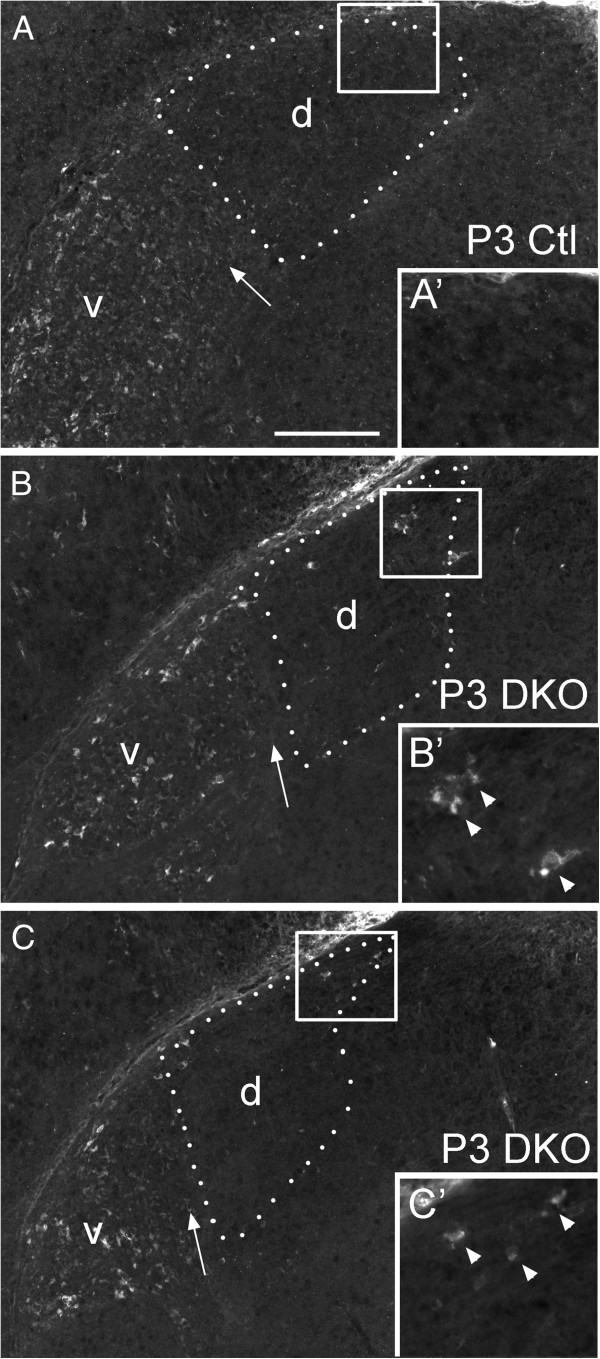
**Ectopic distribution of reelin in *vldlr***^***−/−***^**;*lrp8***^***−/− ***^**mutant LGN.** Reelin distribution was assessed by immunostaining **(A)** P3 control and **(B,C)***vldlr*^*−/−*^;*lrp8*^*−/−*^ mutant (DKO) LGN. Insets show high magnification images of the areas indicated by boxes. Note in (**B**,**C**), the presence of reelin-immunoreactivity in the dorsomedial pole of DKO mutant dLGN. For all images, the outline of the dLGN is depicted with white dots. dLGN are labeled ‘d’, vLGN are labeled ‘v’ and arrows indicate IGL. Scale bar = 180 μm for (**A** to **F**) and 50 μm for insets. DKO, double knockout; dLGN, dorsal LGN; IGL, intergeniculate leaflet; LGN, lateral geniculate nucleus; vLGN, ventral LGN.

These data suggest that classes of IGL projecting retinal axons may target the dorsomedial pole of *vldlr*^*−/−*^;*lrp8*^*−/−*^ mutant dLGN due to the presence of malpositioned IGL neurons. Based on our previous findings that indicated reelin was a class-specific targeting cue for these axons, we next addressed whether reelin distribution was altered in *vldlr*^*−/−*^;*lrp8*^*−/−*^ mutants. Reelin immunostaining was performed on coronal sections of P3 *vldlr*^*−/−*^;*lrp8*^*−/−*^ mutant and littermate control brains. In every mutant examined (n = 3) reelin-expressing cells were observed in the dorsomedial pole of dLGN, as well as in regions of dLGN directly adjacent to the optic tract (Figure 8).

## Discussion

**Figure 9:**
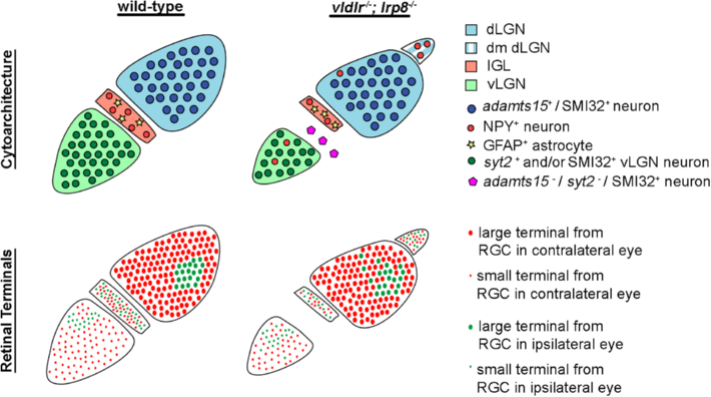
**VLDLR and LRP8 contribute to the cytoarchitecture and retinal targeting of mouse LGN.** A diagram summarizing defects in cytoarchitecture and retinal terminal distribution in mutant mice lacking both VLDLR and LRP8. dmdLGN, dorsomedial pole of dLGN; dLGN, dorsal LGN; IGL, intergeniculate leaflet; LGN, lateral geniculate nucleus; LRP8, low-density lipoprotein receptor-related protein 8; RGC, retinal ganglion cell; VLDLR, very-low-density lipoprotein receptor; vLGN, ventral lateral geniculate nucleus.

At the onset of these studies we hypothesized that deletion of both VLDLR and LRP8 would result in defects in retinogeniculate targeting that phenocopied those observed in *reln*^*rl/rl*^ mutants. At first glance at least three features of retinal targeting in the LGN subnuclei appeared similar in both sets of mutant mice. The size of retinal projections to vLGN appeared reduced in *vldlr*^*−/−*^;*lrp8*^*−/−*^ mutants (Figure 2B,C), a defect previously noted in mutants lacking either reelin or functional DAB1 (Figure 1B) [31]. In all of these cases it remains unresolved if these defects reflect reduced numbers of retinal axons targeting vLGN, smaller terminal arbors of these axons, or smaller retino-recipient nuclei. We also observed an absence of retinal arbors in and adjacent to the IGL in *vldlr*^*−/−*^;*lrp8*^*−/−*^ mutants. Similar uninnervated regions of LGN were observed in all reeler mutants examined and were present in some, but not all, DAB1 mutants [31]. Despite outward similarities in these two phenotypes in *vldlr*^−/−^;*lrp8*^−/−^, *reln*^*rl/rl*^ and *dab1*^*scm/scm*^ mutant LGN, it is important to point out some minor differences. First, the penetrance and strength of these two phenotypes were reduced in receptor mutants compared with those observed in mutants lacking reelin (Table 1). The penetrance and strength was similarly reduced in *dab1*^*scm/scm*^ mutants [31] (unpublished data); however, a small amount of residual DAB1 protein remains present in these spontaneously generated mutant mice raising the question of whether penetrance issues arise from incomplete removal of DAB1. Second, in contrast to *reln*^*rl/rl*^ mutants, an organized set of projections to IGL remained present (albeit reduced) in *vldlr*^*−/−*^;*lrp8*^*−/−*^ mutants (compare IGL in Figure 1B and [31] with those in Figures 2B,C). Third, cytoarchitectural analysis revealed differences in the molecular and cellular make-up of LGN subnuclei in *vldlr*^*−/−*^;*lrp8*^*−/−*^ and *reln*^*rl/rl*^ mutants, a feature that complicates comparisons of the retinogeniculate projection phenotypes described above. Specifically, our studies demonstrated that SMI32-IR neurons abnormally invaded IGL in the absence of VLDLR and LRP8 (Figure 6), a phenotype not observed in mutants lacking reelin [31]. Additionally NPY neurons, which normally reside only in the IGL, migrate into the vLGN and dLGN in the absence of VLDLR and LRP8 but not in the absence of reelin (Figure 7) [31]. These results are summarized in Figure 9.

The final similarity in abnormal retinal projections in *vldlr*^*−/−*^;*lrp8*^*−/−*^ and *reln*^*rl/rl*^ mutants is an incomplete refinement of retinal terminals into eye-specific domains (Figure 9), an essential feature in the establishment of visual system circuits [1,42]. Impaired refinement of retinal projections was also observed in *dab1*^*scm/scm*^, *vldlr*^−/−^, and *lrp8*^−/−^ mutants (Figure 1C,D and data not shown). Since refinement of eye-specific arbors requires retinal activity, defects in eye-specific segregation is not entirely unexpected in these mutants. Retinal activity has previously been shown to be perturbed in the absence of reelin, DAB1, VLDLR or LRP8 [43,44].

Despite the above described similarities in retinal projections in *vldlr*^*−/−*^;*lrp8*^*−/−*^ and *reln*^*rl/rl*^ mutants, two more notable differences in phenotypes were observed in our studies. First, the deletion of both canonical reelin receptors largely failed to generate bundles of misrouted retinal axons that exited the medial borders of the vLGN and IGL, and invaded non-retino-recipient regions of thalamus. Misrouting of axons from ipRGCs into medial thalamus was a distinguishing feature of retinal targeting in the absence of either reelin or DAB1 [20,31]. The lack of ipRGC axons erroneously exiting the medial border of IGL in *vldlr*^*−/−*^;*lrp8*^*−/−*^ mutants may help explain why projections to the IGL in these double mutants appears more developed than in *reln*^*rl/rl*^ mutants. Second, an ectopic region of binocular retinal input was observed in the dorsomedial pole of *vldlr*^*−/−*^;*lrp8*^*−/−*^ LGN (Figure 9), a feature never observed in mutants lacking reelin or functional DAB1 [31]. Cytoarchitectural analyses demonstrated that NPY-expressing IGL neurons aberrantly migrate into this region of *vldlr*^*−/−*^;*lrp8*^*−/−*^ dLGN, and despite being displaced these cells generate a micro-domain that is uniquely distinct from surrounding dLGN and dorsal thalamus. Based on the density of NeuN-positive neurons in this micro-domain we suspect that additional types of non-NPY expressing classes of IGL neurons may also be present in this ectopic region. Certainly, reelin-expressing cells, which normally populate both IGL and vLGN [31], were present in this micro-domain of mutant dLGN. In wild-type tissue, a cohort of glial fibrillary acidic protein (GFAP)-expressing astrocytes also populate IGL but not the surrounding tissue [45]. Surprisingly, we failed to observe GFAP-immunoreactivity in the dorsomedial pole of *vldlr*^*−/−*^;*lrp8*^*−/−*^ mutant dLGN, suggesting that these cell populations were less affected by the loss of VLDLR and LRP8 (data not shown), and hinting that these glial cells are not the source of reelin in the IGL. Based on the presence of IGL target neurons and reelin in the dorsomedial pole of mutant dLGN, we posit that IGL-projecting classes of retinal axons are misrouted into this micro-domain of mutant dLGN. In support of this hypothesis, retinal arbors in this micro-domain remain unsegregated based on their eye of origin and form small terminals, two features that are hallmarks of retinal projections in wild-type IGL. Taken together, data presented here paint a picture that VLDLR and LRP8 have reelin-independent roles in the migration of IGL neurons during thalamic development. Likewise, the lack of misrouted ipRGC axons into medial thalamus suggests that reelin signals through other receptors for the establishment of class-specific retinogeniculate circuits.

What might these non-canonical reelin receptors be? Despite the plethora of studies on reelin’s function in neural development and synaptic function few receptors other than VLDLR and LRP8 have been identified. Integrin α3β1 binds to the N-terminal f-spondin domain of reelin and can activate DAB1 in a reelin-dependent manner [46,47]. Since disruption in α3β1 signaling does not result in severe defects in neuronal position (like deletion of reelin or DAB1), it has been suggested that reelin-α3β1 integrin interactions may be more essential during neurite outgrowth, synapse formation or synaptic function [47-49]. Based on its established role in neurite extension [50,51] and expression in retinal axons [52], α3β1 integrin is a prime candidate to mediate the class-specific axon targeting function of reelin in mouse LGN. However, another prime candidate for mediating reelin’s role in axon targeting in the visual system is amyloid precursor protein (APP), a transmembrane receptor with established roles in mediating neurite outgrowth [53,54] and synapse assembly [55]. APP binds to the central repeating domain of reelin (reelin repeats 3 to 6), an interaction that promotes neurite outgrowth [56]. These findings, together with the fact that APP is expressed by at least some mammalian RGCs [57], make APP a reasonable candidate for mediating the class-specific axon targeting function of reelin in LGN. The last non-canonical reelin receptor that we shall discuss is the protocadherin cadherin-related neuronal receptor 1 (CNR1) [58]. CNR1 remains a controversial reelin binding partner [59,60], but due to isoform diversity and alternative splicing the cadherin superfamily (which includes classical cadherins, protocadherins, FAT-family cadherins, T-cadherins and 7TM-cadherins) [58] has been of particular interest for its role in the specific wiring of neural circuits [61,62]. Based upon the expression of many cadherins and protocadherins by RGCs, and the role of classical cadherins in RGC axon targeting [63-66], CNR1 is an intriguing candidate receptor for mediating the class-specific axon targeting function of reelin in LGN.

Finally, what non-reelin ligand might bind and activate VLDLR and LRP8 to affect neuronal migration during thalamic development? Since both VLDLR and LRP8 are members of the LDL family of lipoprotein receptors they can bind a variety of lipoproteins including compounds containing apolipoprotein E (ApoE). ApoE has been shown to affect cell migration in a variety of systems [67], suggesting it may be the LGN ligand for VLDLR and LRP8. However, other candidate extracellular ligands with known roles in cell migration have also been identified. Both canonical reelin receptors are also capable of binding thrombospondin (THBS), a bulky extracellular proteoglycan present in the developing brain [68,69]. Like reelin, binding of THBS to VLDLR and LRP8 activates DAB1, but the subsequent downstream signaling events differ from that of reelin induced signals [68]. Our previous studies identified an isoform of THBS, THBS4, as being one of the extracellular cues most significantly enriched in vLGN/IGL compared with dLGN at perinatal ages of mouse development [31]. Roles for thrombospondins in LGN development remain unresolved.

## Conclusions

Identifying the molecular underpinnings of class-specific targeting of axons to correct brain regions is essential for our understanding of how complex neural circuits are assembled. Visual system circuits are ideal models to study class-specific axonal targeting since there are over 20 distinct classes of RGCs in the mammalian retina, each with a unique stereotyped pattern of axonal projections to a cohort of retino-recipient nuclei within the brain [11-19,70-73]. Such diversity of neuronal subtypes and class-specific circuitry is not unique to the retina, but instead is a central feature of many regions of the mammalian brain. In a previous study we identified reelin, a bulky extracellular proteoglycan, as a critical molecular component required for the targeting of LGN subnuclei by distinct classes of retinal axons. Here we addressed whether this function of reelin required the two canonical reelin receptors, VLDLR and LRP8. While genetic deletion of either VLDLR or LRP8 had little affect on the initial targeting of retinal axons to LGN subnuclei (despite clear affects on the refinement of these axons into segregated, eye-specific domains), several defects were observed in retinal axon targeting in mutant mice lacking both receptors. However, in contrast to our hypothesis that reelin would utilize these receptors for class-specific retinogeniculate targeting, we found that deletion of both canonical reelin receptors failed to accurately phenocopy retinal targeting defects present in *reln*^*rl/rl*^ mutant LGN. The misrouting of retinal axons into medial, non-retino-recipient thalamus in the absence of reelin were not observed in *vldlr*^*−/−*^;*lrp8*^*−/−*^ mutants and aberrant patterns of retinal projections seen in *vldlr*^*−/−*^;*lrp8*^*−/−*^ mutants (which appeared secondary to defects in neuronal migration) were not observed in mutants lacking reelin. We take these results to suggest that non-canonical reelin receptors contribute to reelin’s role in retinogeniculate targeting, and other extracellular ligands activate VLDLR and LRP8 for the proper migration of IGL neurons.

## Methods

### Mice

Wild-type C57 mice were obtained from Charles River (Wilmington, MA, USA). Reeler mutant mice (*reln*^rl/rl^), *vldlr*^*−/−*^ and *lrp8*^*−/−*^mice were obtained from The Jackson Laboratory (Bar Harbor, ME, USA). Genomic DNA was isolated from tail and genotyping performed as previously described [31,74-76]. The following primer pairs were used: *reln*, TTA ATC TGT CCT CAC TCT GCC CTC T and GCA GAC TCT CTT ATT GTC TCT AC; mutant *reln*, TTA ATC TGT CCT CAC TCT GCC CTC T and TTC CTC TCT TGC ATC CTG TTT TG [73]; *vldlr*, TGG TGA TGA GAG GCT TGT ATG TTG TC and TTG ACC TCA TCG CTG CCG TCC TTG; mutant *vldlr*, CGG CGA GGA TCT CGT CGT GAC CCA and GCG ATA CCG TAA AGC ACG AGG AAG; *lrp8*, CCA CAG TGT CAC ACA GGT AAT GTG and ACG ATG ACC CCA ATG ACA GCA GCG; mutant *lrp8*, GAT TGG GAA GAC AAT AGC AGG CAT GC and GCT TGT TGG AAT TCA GCC AGT TAC C. All analyses conformed to National Institutes of Health (NIH) guidelines and protocols, approved by the Virginia Polytechnic Institute and State University, and Virginia Commonwealth University (VCU) Institutional Animal Care and Use Committees.

### Antibodies

Antibodies for the following antigens were purchased: rabbit anti-VGluT2 and rabbit anti- VGluT1 (diluted 1:500; Synaptic Systems, Göttingen, Germany), mouse anti-reelin (diluted 1:1000; Abcam, Cambridge, UK), rabbit anti-NPY (diluted 1:500; ImmunoStar, Hudson, WI, USA), mouse anti-NeuN (diluted 1:200; Millipore, Billerica, MA, USA), rabbit anti-glutamate decarboxylase 65/67 (GAD65/67) (diluted 1:500; Millipore Bioscience Research Reagents, Temecula, CA, USA), and mouse anti-SMI32 (diluted 1:500; Covance, Princeton, NJ, USA). Fluorescently conjugated secondary antibodies were purchased from Invitrogen (Carlsbad, CA, USA) or Jackson ImmunoResearch (diluted 1:1000; West Grove, PA, USA).

### Immunohistochemistry

Immunohistochemistry (IHC) was performed on 16 μm coronal cryosectioned tissues as previously described [31,38,74,76]. Briefly, tissue slides were allowed to air dry for 15 minutes before being incubated with blocking buffer (2.5% normal goat serum, 2.5% bovine serum albumin and 0.1% Triton X-100 in PBS) for 30 minutes. Primary antibodies were diluted in blocking buffer and incubated on tissue sections for overnight at 4°C. On the following day, tissue slides were washed in PBS and secondary antibodies diluted 1:1000 in blocking buffer were applied to slides for 1 hour at room temperature. After thoroughly washing in PBS, tissue slides were coverslipped with VectaShield (Vector Laboratories, Burlingame, CA, USA). Images were acquired on a Zeiss Axio Imager A2 fluorescent microscope, a Zeiss Examiner Z1 LSM 710 confocal microscope, or a Zeiss LSM 700 confocal microscope (Oberkochen, Germany). When comparing different ages of tissues or between genotypes, images were acquired with identical parameters. The size of VGluT2-immunoreactive retinal terminals was measured with ImageJ (NIH, Bethesda, MD, USA). The area of individual puncta in control and mutant vLGN, IGL and dLGN were measured manually. A minimum of three animals (per genotype and per age) was compared in all IHC experiments.

### *In situ* hybridization

*In situ* hybridization (ISH) was performed on 16 μm coronal cryosectioned tissues as previously described [31,74,76]. The generation of *adamts15* (clone IDs 30619053) riboprobes was previously described [31]. Briefly, riboprobes were synthesized using digoxigenin (DIG)-labeled UTP (Roche, Mannheim, Germany) and the MAXIscript In Vitro Transcription Kit (Ambion, Austin, TX, USA). Probes were hydrolyzed to 500 nt. Coronal brain sections were prepared and hybridized at 65°C as previously described (Su *et al*., 2010), and bound riboprobes were detected by horseradish peroxidase (POD)-conjugated anti-DIG antibodies and fluorescent staining with Tyramide Signal Amplification (TSA) systems (PerkinElmer, Shelton, CT, USA). Images were obtained on a Zeiss Axio Imager A2 fluorescent microscope or a Zeiss Examiner Z1 LSM 710 confocal microscope. A minimum of three animals per genotype and age was compared in ISH experiments.

### Intraocular injections of anterograde tracers

Intraocular injection of CTB conjugated to Alexa Fluor 488 or Alexa Fluor 594 (Invitrogen) was performed as previously described [31,39]. Briefly, mice were anesthetized with hypothermia (<P7) or by isoflurane vapors (>P7). The sclera was pierced with a sharp-tipped glass pipette and excess vitreous was drained. Another pipette, filled with a 0.1 to 0.2% solution of CTB, was inserted into the hole made by the first pipette. The pipette containing the CTB was attached to a picospritzer and a prescribed volume (1 to 3 μl at P3 to P10 and 3 to 5 μl for ages >P10) of solution was injected into the eye. After 1 to 2 days, mice were killed and brains were fixed in 4% paraformaldehyde. A total of 100 μm coronal sections were sectioned on a vibratome (Microm HM 650 V; Thermo Scientific, Waltham, MA, USA) and mounted in ProLong Gold (Invitrogen). Retinal projections were analyzed from between 3 to 12 animals for each age and genotype. Images were acquired on a Zeiss Examiner Z1 LSM 710 confocal microscope or a Zeiss LSM 700 confocal microscope. We documented four types of defects in retinogeniculate targeting in wild-type (control), *reln*^*rl/rl*^*, vldlr*^*−/−*^*, lrp8*^*−/−*^*, vldlr*^*−/−*^;*lrp8*^*−/−*^*, vldlr*^*−/−*^;*lrp8*^*+/−*^ and *vldlr*^*+/−*^;*lrp8*^*−/−*^ mice: 1) smaller pattern of retinal projection to vLGN; 2) an absence of retinal axons between vLGN and IGL; 3) retinal axons abnormally exiting the medial border of the vLGN with IGL; and 4) retinal axons from both ipsilateral and contralateral retinas that ectopically innervate to the dorsomedial pole of dLGN. We scored these phenotypes objectively based on penetrance and subjectively based on robustness. All sections were scored blind by at least two observers.

## Abbreviations

ADAMTS15: A disintegrin and metalloproteinase with thrombospondin type 1 motif member 15; ANOVA: Analysis of variance; ApoE: Apolipoprotein E; ApoER2: Apolipoprotein E receptor 2; APP: Amyloid precursor protein; CNR1: Cadherin-related neuronal receptor 1; CNS: Central nervous system; CT: Corticothalamic; CTB: Cholera toxin B subunit; DAB1: Disabled-1; DIG: Digoxigenin; DKO: Double knockout; dLGN: Dorsal LGN; dmdLGN: Dorsomedial pole of dLGN; EGF: Epidermal growth factor; GAD: Glutamate decarboxylase; GFAP: Glial fibrillary acidic protein; IGL: Intergeniculate leaflet; IHC: Immunohistochemistry; ipRGC: Intrinsically photosensitive RGC; ISH: *In situ* hybridization; LDL: Low-density lipoprotein; LGN: Lateral geniculate nucleus; LRP8: Low-density lipoprotein receptor-related protein 8; NeuN: Neuronal nuclei; NIH: National Institutes of Health; NPY: Neuropeptide Y; PBS: Phosphate-buffered saline; POD: Peroxidase; RGC: Retinal ganglion cell; SMI32-IR: SMI32-immunoreactivity; syt2: Synaptotagmin 2; THBS: Thrombospondin; TSA: Tyramide Signal Amplification; VCU: Virginia Commonwealth University; VGluT1: Vesicular glutamate transporter 1; VGluT2: Vesicular glutamate transporter 2; VLDLR: Very-low-density lipoprotein receptor; vLGN: Ventral LGN.

## Competing interests

The authors declare that they have no competing interests.

## Authors’ contributions

JS carried out the collection, preparation and imaging of CTB injected tissues, performed IHC and ISH experiments, and helped draft portions of the manuscript. MAK carried out data collection and image analysis. AMJ carried out the collection, preparation and imaging of CTB injected tissues. MAF conceived, designed and coordinated the study, and drafted the manuscript. All authors read and approved the final version of the manuscript.
